# Potential of activated microglia as a source of dysregulated extracellular microRNAs contributing to neurodegeneration in amyotrophic lateral sclerosis

**DOI:** 10.1186/s12974-020-01822-4

**Published:** 2020-04-28

**Authors:** Eleni Christoforidou, Greig Joilin, Majid Hafezparast

**Affiliations:** School of Life Sciences, University of Sussex, Falmer, Brighton, BN1 9QG UK

**Keywords:** Microglia, microRNA, Amyotrophic lateral sclerosis, Neurodegeneration

## Abstract

Amyotrophic lateral sclerosis (ALS) is the most common form of motor neuron degeneration in adults, and several mechanisms underlying the disease pathology have been proposed. It has been shown that glia communicate with other cells by releasing extracellular vesicles containing proteins and nucleic acids, including microRNAs (miRNAs), which play a role in the post-transcriptional regulation of gene expression. Dysregulation of miRNAs is commonly observed in ALS patients, together with inflammation and an altered microglial phenotype. However, the role of miRNA-containing vesicles in microglia-to-neuron communication in the context of ALS has not been explored in depth. This review summarises the evidence for the presence of inflammation, pro-inflammatory microglia and dysregulated miRNAs in ALS, then explores how microglia may potentially be responsible for this miRNA dysregulation. The possibility of pro-inflammatory ALS microglia releasing miRNAs which may then enter neuronal cells to contribute to degeneration is also explored. Based on the literature reviewed here, microglia are a likely source of dysregulated miRNAs and potential mediators of neurodegenerative processes. Therefore, dysregulated miRNAs may be promising candidates for the development of therapeutic strategies.

## Introduction

Amyotrophic lateral sclerosis (ALS) is the most severe and most common form of motor neuron degeneration in adults, with an estimated worldwide prevalence of 5 cases per 100,000 population and an incidence of approximately 2 per 100,000 individuals per year [1]. It targets the motor cortex, brainstem and spinal cord and involves the death of upper and lower motor neurons that control voluntary muscles. This results in symptoms such as muscle stiffness and twitching, limb weakness due to a gradual decrease in muscle size, and difficulty swallowing or speaking. Additionally, up to half of the patients develop frontotemporal dementia (FTD) [2] which is characterised by progressive degeneration in the frontal and temporal lobes, behavioural and personality changes and language and executive function decline [3, 4]. In most cases, during the late stages of the disease, the weakening of the diaphragm and intercostal muscles results in death by respiratory failure [5]. ALS is a heterogeneous disease in age of onset and progression rate, with a median survival time of 3 to 5 years from diagnosis [6].

Around 10% of ALS cases are due to inherited genetic mutations (familial ALS; fALS). These cases are associated with over 25 genes, of which the most common are chromosome 9 open reading frame 72 (*C9orf72*) [7], superoxide dismutase 1 (*SOD1*) [8], fused in sarcoma (*FUS*) [9] and transactive response DNA-binding protein 43 (*TARDBP; TDP-43)* [10]. The remaining 90% of cases are sporadic (sALS) and arise without any family history, but around 10% of patients with sALS have genetic mutations like those in fALS [11].

Motor neurons and surrounding oligodendrocytes in ALS spinal cord, cerebellum, hippocampus, and frontal and temporal cortices have characteristic protein-rich cytoplasmic inclusions and aggregates [12, 13]. The most common are ubiquitinated-protein aggregates known as Lewy body-like or skein-like inclusions, characterised by randomly orientated filaments covered by fine granules. TDP-43, which is predominantly found in the nucleus, is hyper-phosphorylated, cleaved and mislocalised into these cytoplasmic inclusions in most sALS cases and most SOD1-negative fALS patients [14–19]. FUS-positive inclusions are similarly mislocalised to the cytoplasm and are also observed in the spinal cord of sALS and SOD1-negative fALS patients [20–22]. In *FUS* mutation carriers, there is a normal TDP-43 distribution, but FUS-positive cytoplasmic inclusions are observed in lower motor neurons [20], whereas in *SOD1* mutation carriers, SOD1 protein aggregates are observed [23, 24]. The exact pathological effects of these inclusions remain largely unknown, and whether mislocalisation leads to impaired cellular function due to the proteins being sequestered and unable to function properly is unclear.

Several mechanisms underlying the disease-mediated toxicity seen in fALS have been proposed such as glutamate excitotoxicity [25], endoplasmic reticulum (ER) stress [26–28], inhibition of the proteasome [29, 30], mitochondrial damage [31–33], extracellular toxicity of misfolded proteins [34, 35], aberrant superoxide production [36], microhaemorrhages of spinal capillaries [37] and axonal disorganisation and disrupted axonal transport [38–41]. Furthermore, the identification of mutations in the *FUS* and *TARDBP* genes, which are involved in the production of proteins that take part in RNA splicing, translation, transport and microRNA (miRNA) biogenesis, suggests a potential role of altered RNA expression and metabolism in the disease [42]. Indeed, post-mortem pathological analysis of both fALS and sALS cases has shown the presence of abnormal levels of RNA and RNA-binding proteins in both motor neurons and glial cells [12].

Although neurodegenerative diseases were traditionally considered as having cell autonomous mechanisms (i.e. damage within a population of neurons being enough to cause disease), the death of motor neurons in ALS is influenced by non-neuronal cells such as astrocytes and microglia [43–45], and non-cell-autonomous mechanisms appear to play significant roles in the disease onset and/or progression. In fact, motor neuron degeneration appears to be dependent on neighbouring glia expressing mutant proteins [46, 47]. For example, studies have shown that high expression of mutant SOD1 in either most or all motor neurons of mice is insufficient for disease onset [46, 47], whereas mutant SOD1 expressed within microglia is required for disease to occur [48, 49]. Additionally, extracellular mutant SOD1 from the *SOD1*^G93A^ mouse model of ALS does not cause detectable direct killing of motoneurons in culture, but it activates microglia which then release toxic factors that lead to motor neuron death [35]. In line with this, reducing the expression of mutant SOD1 within microglia slows disease progression [43]. Similarly, deletion of mutant *SOD1* within astrocytes, oligodendrocytes and NG2 glial cells delays disease progression and improves survival [44, 50, 51]. Therefore, although ALS was once considered a motor neuron disease, it is now known as a multi-cellular and multi-systemic disease [52, 53], with motor neuron death being primarily driven by glial cell pathology as well as a convergence of other damaging mechanisms such as inflammatory conditions [54, 55].

Recently, it has been documented that glia also communicate with other cells by releasing extracellular vesicles containing proteins and nucleic acids, including miRNAs. However, the involvement of miRNA-containing vesicles in microglia-to-neuron communication in the context of ALS has not been explored in depth. Hence, this review will first summarise the evidence for the presence of inflammation, pro-inflammatory microglia and dysregulated miRNAs in the disease, then explore how microglia may potentially be responsible for this miRNA dysregulation by presenting evidence for a coexistence of an altered miRNA expression and a neurodegeneration-related microglial state in ALS. Finally, the possibility of pro-inflammatory ALS microglia releasing dysregulated miRNAs, which may then enter neuronal cells to cause degeneration, will be explored by reviewing studies that show how glia-to-neuron transfer of nucleic acids leads to functional changes within recipient neurons.

## Inflammation is present in ALS

The levels of several pro-inflammatory cytokines are altered in ALS, suggesting the presence of inflammation. For example, an increase in the protein expression levels of tumour necrosis factor (TNF), interleukin (IL)-8, IL-12, IL-17(A), interferon (IFN)-γ and monocyte chemoattractant protein (MCP)-1 in the blood serum and/or cerebrospinal fluid (CSF) of ALS patients has been observed by at least two independent studies [56–62]. Moreover, increased expression of several chemokines was found in the CSF of ALS patients [62]. Although the expression of IL-1β, IL-2, IL-6 and IL-15 has been found upregulated in the serum or CSF of ALS patients in some studies, it remained unchanged in other studies [56, 57, 59–65], likely due to the different detection methods employed. Furthermore, increased production of TNF and IL-1β, as well as reactive oxygen species (ROS) and prostanoids, was observed in spinal cord tissue from ALS patients [66]. It has also been shown that chronic administration of IL-1β results in neurodegeneration [67], whereas IL-1β depletion or IL-1 receptor antagonism attenuates inflammation and prolongs the lifespan of ALS mouse models [68], providing further evidence of the importance of inflammation in the pathology of ALS. Moreover, increased levels of lymphocyte function-associated molecule 1 (LFA-1) and leukocyte common antigen (LCA), which are found on lymphocytes, suggest an infiltration of peripheral immune cells into the central nervous system (CNS). Increased levels of complement receptors CR3 and CR4 [69, 70], which are mostly expressed on monocytes and macrophages, as well as increased mRNA and protein levels of the complement components C1q, C3, C4 and C5b-9, have also been reported in the spinal cord and motor cortex of ALS patients [71], suggesting increased inflammation and phagocytosis in these areas. Additionally, upregulation of cyclooxygenase 2 (COX2), a common target of anti-inflammatory drugs that normally contributes to memory consolidation, synaptic activity and functional hyperaemia, is observed in ALS tissues, again suggesting an increase in neuroinflammation [72].

Changes in glial cells also play a role in ALS inflammation. For example, increased expression of Toll-like receptor 4 (TLR4) mRNA and protein is detected in astrocytes in both the grey and white matter of the spinal cord in ALS, whereas increased expression of TLR2 mRNA and protein is observed in microglia [73]. Significant upregulation of TLR7 (the murine orthologue of human TLR8) mRNA was observed in the anterior lumbar spinal cord of ALS patients, together with an increase in the number of astrocytes and activated microglia [65]. These receptors constitute some of the “sensors” of the immune system and mediate the continued glial reactivity seen in pathological conditions [74]. Furthermore, increased IFN-γ immunoreactivity, a cytokine important for macrophage activation and induction of major histocompatibility complex (MHC) class II molecule expression on immune cells, is observed in glia and neurons in the ventral horn of the spinal cord in ALS, compared to controls [75], suggesting a sustained activation of the innate immune response. Therefore, a plethora of studies confirm the presence of inflammation in ALS, even though ALS is not considered an inflammatory disease.

## Inflammation in ALS involves the activation of microglia

Microglia are distributed throughout the brain and spinal cord parenchyma and account for 10–20% of the total glial population [76, 77]. They are the main defence cells in the CNS against invading bacteria, viruses and prions [78, 79], and they are responsible for maintaining brain homeostasis [80]. They are phagocytic cells that secrete several pro-inflammatory cytokines [80] and other neurotoxic substances such as nitric oxide (NO) [81] and reactive oxygen intermediates [82]. Under homeostatic conditions, microglia display surveillance behaviour, evidenced by their low mobility, small cell bodies and extensive, highly motile processes constantly scanning the CNS environment [83, 84]. This “resting” state is commonly referred to as the neutral or “M0” state [85] and is characterised by a low expression of macrophage-related surface markers, such as CD45 and MHC II [77].

Microglia are extremely sensitive to physiological changes in their environment and become “activated” following exposure to specific cytokines and growth factors that indicate infection, trauma, neuronal insult or inflammation [77]. Exposure to pro-inflammatory cytokines induces “classical” activation of microglia and switching to a pro-inflammatory phenotype or “M1” state, which is neurotoxic [86]. On the other hand, exposure to anti-inflammatory cytokines such as IL-4 induces “alternative” activation and switching to an anti-inflammatory phenotype or “M2” state which promotes tissue repair [85]. Interestingly, M1- and M2-associated genes can be co-expressed [87], suggesting that the two states are not mutually exclusive and that this binary classification is insufficient.

Increased numbers of activated microglia have been observed in the CNS of ALS mouse models and human ALS patients [88, 89]. Moreover, studies on post-mortem tissues from human patients and ALS mouse models have shown increased levels of activated microglia in areas of the brain with neuronal loss [43, 90]. For example, extracellular ATP binding to the P2X7 receptor on microglia is known to induce a pro-inflammatory response [91], and the P2X7 receptor was found elevated in the microglia of ALS patients [92]. Activation of this receptor by the agonist BzATP (2′3′-O-(benzoyl-benzoyl) ATP) in primary microglia from *SOD1*^G93A^ mice enhances production of several pro-inflammatory mediators which may lead to neuronal degeneration [93–95]. It was also shown that the level of microglial activation parallels motor neuron degeneration in ALS patients [96, 97], and that microglia expressing mutant *SOD1*^G93A^ in mice are more activated than wild-type microglia [98]. Furthermore, mice overexpressing *SOD1* show an increase in M1-like microglia [99], and *SOD1*^G93A^ mutations in rat microglia result in accelerated disease progression, compared to wild-type microglia [100].

The transcription factor NF-κB (nuclear factor kappa-light-chain-enhancer of activated B cells) is induced by certain pro-inflammatory cytokines and regulates genes responsible for the innate and adaptive immune response. Several studies have shown that this transcription factor is upregulated in glial cells of both sALS and fALS patients [101–103]. Interestingly, the ALS-associated gene optineurin negatively regulates pro-inflammatory-mediated NF-κB activation [104], and loss-of-function mutations in this gene are seen in some ALS patients [102]. Consistent with this, *SOD1*^G93A^ mice exhibit NF-κB hyperactivation in microglia, while NF-κB inhibition extends the survival of these mice by slowing disease progression [101].

In addition to the altered state of microglia observed in ALS, evidence also suggests microglial degeneration in the disease. For example, mononuclear phagocytes (which include both CNS-resident microglia and infiltrating monocytes from the periphery) were shown to degenerate in transgenic mutant SOD1 rats [105] and mice [106, 107]. A similar degeneration of microglia has also been observed in the brains of Alzheimer’s disease (AD) patients [108]. However, peripheral monocyte infiltration in ALS remains a controversial topic; several lines of evidence suggest that peripheral monocytes invade the CNS in ALS [107, 109, 110], whereas others have shown no contribution of peripheral monocytes to the disease [87, 111]. Therefore, whether degenerating immune cells in ALS include CNS-resident microglia, infiltrating monocytes or both requires further investigation.

A unique population of immune cells has recently been identified and was termed “disease-associated microglia” (DAM). DAM not only express microglial markers such as *Hexb*, *Iba1* and *Cst3*, but also downregulate genes commonly associated with homeostatic microglia, such as *Cx3cr1*, *P2ry12/13* and *Tmem119*. DAM also upregulate the expression of neurodegeneration-specific genes, such as *Trem2*, *Apoe*, *Tyrobp*, *Lpl* and *Ctsd* [112]. This transcriptionally distinct microglial population has primarily been associated with AD mouse models [112–116], but a DAM-like phenotype has also been observed in ageing [112, 116–118], tauopathy models [119] and ALS [87, 112, 113, 116, 120]. It has therefore been proposed that DAM are not part of a specific disease aetiology, but are rather a common occurrence following CNS pathology [121]. The transition of homeostatic microglia to DAM, their role in health and disease and the potential impact of their discovery in the development of therapies have previously been discussed [121, 122] and are beyond the focus of this review. Interestingly, at least in AD, the role of DAM may be protective, since gain-of-function mutations in genes downregulated in DAM and loss-of-function mutations in genes upregulated in DAM are associated with an increased risk of developing AD [121]. Nonetheless, whether DAM have a similarly protective role in ALS is unclear and warrants further investigation. In conclusion, there is overwhelming evidence that the inflammation seen in ALS involves the pro-inflammatory activation of microglia, and an abundance of research suggests the presence of transcriptionally distinct microglia in ALS.

## Causes of microglial activation in ALS

Several hypotheses as to how microglial activation occurs in ALS have been proposed. Studies suggest that exposure to low levels of systemic signalling molecules associated with ageing and chronic inflammation (i.e. microglial “priming”) can exacerbate the microglial response to a second local stimulus, such as the presence of protein aggregates characteristic of neurodegenerative diseases, potentiating tissue damage [123]. Additionally, it was recently shown that IL-1β-mediated activation of astrocytes overexpressing wild-type FUS alter their cross-talk with microglia so that microglia acquire a pro-inflammatory profile resembling the phenotype seen in ALS [124]. It was further suggested that the mechanism of this activation involved an increase in the level of prostanoids released by these astrocytes; however, no evidence was found to support this. Instead, other pro-inflammatory cytokines such as IL-5, IL-6, IL-7, IL-15 and other molecules under astrocytic NF-κB transcriptional control have been suggested as likely candidates driving microglial activation [124]. It was also proposed that T-cells interacting with microglia may cause their activation, since spinal cord infiltration of T_h_ and T_c_ cells increases over time in ALS mice, compared to controls. Furthermore, expression of humoral immune response, oxidative phosphorylation and ROS genes in microglia was found to correlate with T_h_ cell numbers, whereas expression of genes involved in phagocytosis and coenzyme metabolism in microglia was correlated with T_c_ cell numbers [87].

Another hypothesis is that sub-clinical infections that activate the immune system could activate microglia and lead to the inflammation seen in ALS. However, a recent study using RNA sequencing to locate common parasitic and bacterial genomes in areas of activated microglia in CNS tissues from ALS patients found no supporting evidence [125]. Nevertheless, only a limited list of infectious agents was examined, and there are several limitations with the study, such as the possibility of loss of infectious agent transcripts as the disease progresses (since the tissues were from end-stage cases only), as well as RNA decay during frozen sectioning and variable post-mortem intervals that could have interfered with the RNA sequencing. Therefore, the possibility of sub-clinical infections causing chronic microglial activation and neurodegeneration requires further investigation.

Another possible activation route is via changes in gut microbiota, since these can reportedly control the maturation and function of microglia [126]. However, studies linking ALS with alterations in gut microbiota in patients and mouse models (reviewed in [127, 128]) are limited, and even though they suggest some instances of gut dysbiosis, whether microglia are activated in those cases has not been examined.

The most convincing evidence of how microglia may become activated in ALS comes from studies showing that extracellular misfolded proteins such as oxidised or mutant SOD1 secreted from astrocytes or neurons through neuroendocrine pathways can activate microglia and subsequently cause motor neuron death [34, 129, 130]. Additionally, mutant SOD1 proteins are not directly neurotoxic in the absence of microglia [35]. Similarly, five forms of extracellular TDP-43 (TDP-43-WT, TDP-43-M337V, TDP-25-WT, TDP-25-M337V, TDP-43-A315T) can activate microglia, with lower doses of the mutant proteins having a greater activating effect than the wild-type proteins [131]. This activation is the result of TDP-43 proteins interacting with microglial CD14, a pattern recognition receptor that also interacts with mutant SOD1 [35]. Again, in the absence of microglia, extracellular mutant TDP-43 proteins are not neurotoxic. Furthermore, incubation of microglia with both wild-type and mutant TDP-43 causes an increase in phosphorylated p65 (one of the subunits of NF-κB and an index of NF-κB activation) and phosphorylated p38 mitogen-activated protein kinase (MAPK; a mediator of the MAPK pathway that controls responses to stress and cytokines). Additionally, IκB—an inhibitor of NF-κB activation and pro-inflammatory cytokine production—decreases in microglia incubated with wild-type or mutant TDP-43 [131].

Interestingly, upregulation of TDP-43 within microglia can also enhance the microglial response via activation of the NF-κB pathway that results in the release of increased TNF, NOX2, IL-1β, IL-6 and associated ROS and reactive nitrogen species (RNS) that are toxic to neighbouring neurons [103]. TDP-43 may also promote microglial activation through interactions with activator protein 1 (AP-1), a transcription factor regulating gene expression in response to cytokines, stress, growth factors and other stimuli. Indeed, SR11302, an AP-1 inhibitor, was able to block IL-1β protein expression in microglia treated with wild-type TDP-43 [131]. Therefore, it is likely that the microglial activation in ALS is primarily driven by misfolded proteins expressed within the cells or by extracellular mutant proteins targeting microglia. Other mechanisms of microglial activation could exist; however, more studies are needed to examine this.

## Possible mechanisms of microglia-mediated neurodegeneration in ALS

Despite the evidence for the presence of inflammation in ALS, exactly how glia-mediated neuroinflammation contributes to disease progression is still unclear. A possible mechanism of microglia-mediated neurotoxicity is via the production of ROS, RNS and pro-inflammatory cytokines, which may result in tissue injury and neurodegeneration [132–135]. Importantly, increased ROS and NO release from activated microglia is seen in some fALS patients with *SOD1* mutations and correlates with neuronal cell death [136, 137]. Furthermore, some of the soluble factors released by microglia, which alter neuron excitability and affect synaptic function, are also involved in neuroinflammatory disorders. These factors regulate the expression of important molecules for synaptic plasticity such as cofilin or CREB (cAMP response element-binding protein) [138] or modulate the properties and expression of synaptic channels [139–143]. Studies have also shown that microglia regulate neuronal synapses via contact-dependent mechanisms such as synaptic element engulfment, leading to synapse loss during CNS inflammation [144–146]. However, it is likely that microglia-mediated synapse elimination is an appropriate response to remove a “diseased” synapse that is abnormally inactive due to causes unrelated to microglia (for example, because of degenerating neurons).

Glutamate excitotoxicity has also been suggested to play a role in microglia-mediated neurodegeneration in ALS. For example, increased levels of glutamate are detectable in the CSF of some ALS patients [147]. A reduction of extracellular glutamate uptake by astrocytic glutamate transporters has been observed in ALS, and this contributes to motor neuron death [138, 148]. Activated microglia increase the susceptibility of motor neurons to glutamate toxicity, through reducing glutamate uptake by astrocytes [149]. Furthermore, TNF-dependent glutamate release by activated microglia induces cortical neuron death [150], whereas blockade of excessive glutamate release by activated microglia suppresses neuronal loss in the spinal cord of ALS mouse models [151]. Evidence also suggests that microglia expressing mutant SOD1 mediate neuronal death via an overproduction of D-serine, a co-agonist at *N*-methyl-d-aspartate (NMDA) receptors [152]. Furthermore, it has been proposed that activated microglia release increased amounts of quinolinic acid, which may bind to NMDA receptors on motor neurons to cause excitotoxicity [153]. Nevertheless, glutamate excitotoxicity may play only a minor role in ALS pathogenesis since several tested drugs targeting glutamatergic transmission either did not work, or had only a modest effect on life span (reviewed in [154]).

## The levels of certain miRNAs are altered in ALS

MiRNAs are small, approximately 22 nucleotides-long, non-coding RNAs (ncRNAs) transcribed from intergenic regions or from introns of protein-coding genes by RNA polymerases [155] and are involved in the regulation of translation [156]. They bind to complementary mRNA sequences, resulting in gene silencing via degradation of the mRNA or translational repression [157]. The process of mRNA degradation following interaction with a miRNA involves deadenylation and decapping, followed by 5′ to 3′ exonucleolytic digestion [158–160]. In the case of translational repression, the function of the ribosomes during the elongation step can be hindered, and the recognition of the eukaryotic translation initiation factor 4F (eIF4F) cap can be inhibited [161–163]. A single miRNA usually targets many different genes, and often a set of miRNAs synergistically target a single gene [164]. The brain has the highest expression of tissue-specific miRNAs [165–167], with a specific set of them localised to dendrites, where they play a role in adult neuronal plasticity [168, 169] and dendritic spine morphology [170].

The ALS genes *FUS* and *TARDBP* are directly involved in miRNA processing, by enhancing production through Drosha recruitment—a ribonuclease enzyme involved in miRNA biogenesis [171]—and by promoting the interaction between Drosha and Dicer—an RNase endonuclease involved in pre-miRNA processing [172]. Furthermore, TDP-43 protein is normally involved in the post-transcriptional maturation of certain miRNAs in the cytoplasm and nucleus. Consequently, its mislocalisation in cytoplasmic aggregates in ALS has been associated with a decrease in Dicer and Drosha processing of TDP-43-regulated miRNAs [172].

The role of miRNAs in ALS pathology was evidenced when a differential miRNA expression profile was observed between ALS patients and healthy controls in the CSF and blood serum and plasma [107, 173–189], giving rise to the opportunity of using them as potential biomarkers [190, 191]. However, the source of these miRNAs is unknown, and further studies are required to identify if these are released by degenerating motor neurons, atrophied muscles, activated astrocytes and microglia, or other cell types. Researchers have so far investigated astrocytes and neurons as a potential source. Using human patient-induced astrocytes, it was shown that a dysregulated release of 137 miRNAs occurs in culture [192]. However, of the 85 upregulated miRNAs released by these cells, only two have also been found upregulated in the CSF of ALS patients [176, 188] and none correspond to those upregulated in the blood of patients. Similarly, of the 52 downregulated miRNAs released by induced patient astrocytes, only three were also identified as downregulated in the CSF or blood of patients [188, 193]. Therefore, the contribution of astrocytes to extracellular miRNAs in ALS requires further investigation. In terms of neurons as a source of extracellular miRNAs, a recent study identified 30 differentially expressed miRNAs in neuron-derived extracellular vesicles in the plasma of ALS patients [179]. Surprisingly, none of these correspond to previously identified dysregulated miRNAs in plasma [174, 183, 184]. Therefore, the current limited evidence suggests that astrocytes and neurons are an unlikely source of the circulating dysregulated miRNAs in ALS.

In theory, miRNA upregulation within cells may result in their concurrent upregulated release. Therefore, investigating the miRNA expression of specific cell types in ALS tissues may provide a starting point of identifying the source of dysregulated circulating miRNAs. In fact, over 100 miRNAs involved in cell death pathways, inflammation, immune responses and defence responses have been found dysregulated in the spinal cord of ALS patients [194, 195] (summarised in Additional file 1). It was suggested that these changes are primarily due to altered miRNA expression within motor neurons. However, a recent study observed a downregulation of only four miRNAs in motor neurons [196], of which only one was previously found to be downregulated in spinal cord tissue. Similarly, miRNAs downregulated in patient-induced motor neuron progenitors in culture [197] do not correspond to any of those downregulated in biological fluids. Circulating miRNAs may also be released by atrophied muscles during the progression of ALS. Indeed, studies have shown differential miRNA expression in muscle tissue from ALS patients and mouse models, compared to healthy controls ([198–203] and reviewed in [204]). However, only a small percentage of the miRNAs dysregulated in muscles are also differentially expressed in biological fluids (Additional file 1). These findings suggest that cell types other than motor neurons and muscle cells may be responsible for the release of miRNAs in the disease. Furthermore, the cell types responsible for the differential miRNA expression in the spinal cords of patients remain unknown.

## Microglial activation is associated with miRNA dysregulation in ALS

Recent evidence suggests that dysregulation of certain miRNAs is linked specifically to the microglial activation seen in ALS. For example, miR-22-3p, miR-125b-5p, miR-146b-5p, miR-155-5p, miR-214-3p and miR-365-3p are overexpressed in microglia from *SOD1*^G93A^ mice, and these modulate inflammatory genes linked to ALS, such as the IL-6 pathway which determines the transcription of TNF [205]. Moreover, in vitro activation of wild-type microglia results in the upregulation of several miRNAs, including miR-22-3p, miR-125b-5p, miR-146b-5p and miR-155-5p, which are similarly upregulated in *SOD1*^G93A^-overexpressing microglia.

MiR-155-5p overexpression is also seen prior to disease onset and throughout disease progression in spinal cord tissue from both sALS and fALS patients [206], as well as in end-stage *SOD1*^G93A^ rat and mouse spinal cords. This overexpression is accompanied by an elevation of all inflammatory miRNAs, as well as several genes associated with neuroinflammation, astrogliosis and microglial activation [207]. Furthermore, genetic ablation of miR-155-5p in *SOD1*^G93A^ mice decreases the number of resident microglia in the mouse spinal cord and also prolongs survival, enhances performance on motor tasks and delays disease onset [208]. Interestingly, even though microglia have normal phagocytic ability in miR-155-5p^−/−^ mice, *SOD1*^G93A^/miR-155-5p^+/−^ mice produce microglia impaired in their phagocytic ability and thus are unable to clear dead neurons. In parallel, most of the direct gene targets of miR-155-5p are no longer repressed in miR-155-5p^−/−^ mice. Similarly, central administration of anti-miR-155-5p in SOD1 mouse models de-represses miR-155-5p gene targets within neurons, astrocytes and microglia, paralleled by increased survival and improved motor function. These findings are consistent with reports that miR-155-5p is found upregulated in ALS patients and that anti-miR-155-5p extends survival in mice with *SOD1* mutations [207]. Therefore, miR-155-5p appears to play a role in the pathology of ALS through modulating microglial function.

Given the evidence that microglial activation and miRNA dysregulation in ALS co-occur, it is possible that microglia are a possible source of circulating miRNAs found dysregulated in the blood and CSF of ALS patients (Table 1). Nonetheless, no studies have investigated this possibility so far. Furthermore, apart from two studies showing upregulation of certain miRNAs within microglia in ALS mouse models [107, 205], no studies have confirmed whether this is also the case in human patients. Of the 29 miRNAs upregulated in microglia from ALS mouse models, six are also upregulated in the CSF and/or serum of human patients (Table 1). These six miRNAs collectively target over 10,000 experimentally validated genes. Gene ontology analysis of these genes showed enrichment in several biological processes, including processes that are disrupted in ALS, such as cytoskeletal dynamics, oxidative stress, inflammation, RNA regulation and organelle transport (Additional file 2).

**Table 1:** List of miRNAs upregulated in the blood and CSF of ALS patients

Reference	miRNA	Source
[173]	hsa-miR-181a-5p	CSF
[107]	**hsa-miR-146a** hsa-miR-150 **hsa-miR-328** **hsa-miR-532-3p** **hsa-miR-99b**	CSF
[107, 188]	**hsa-miR-27b-3p**	CSF
[174]	hsa-miR-424	Plasma
[174, 183, 185–187]	hsa-miR-206	Serum, plasma
[175]	hsa-miR-338-3p	CSF, serum
[176]	hsa-miR-143-5p hsa-miR-574-5p	CSF
[180]	**hsa-miR-142-3p**	Serum
[181]	hsa-miR-1 hsa-miR-144-5p hsa-miR-192-3p hsa-miR-19a-3p	Serum
[181, 185]	hsa-miR-133a-3p hsa-miR-133b	Serum
[184]	hsa-miR-4649-5p	Plasma
[186]	hsa-miR-106b	Serum
[187]	hsa-miR-143-3p	Serum
[188]	hsa-miR-9-5p hsa-miR-124-3p hsa-miR-125b-2-3p hsa-miR-127-3p hsa-miR-143-3p	CSF

Bold indicates miRNAs that are also upregulated inside spinal cord microglia from *SOD1*^G93A^ mice, according to [107, 205]

## Released miRNAs as a potential mechanism of microglia-mediated neurodegeneration

Another microglia-mediated mechanism of neurotoxicity is likely to be via the release of miRNAs into the extracellular environment. In biological fluids such as blood and CSF, miRNAs can be found in vesicles (exosomes, ectosomes/microvesicles and apoptotic bodies) [209, 210], or they can be bound to proteins that increase their stability in the extracellular space, such as AGO2 [209, 211]. Furthermore, it is known that microglia communicate with neighbouring neurons via the secretion of extracellular vesicles [212, 213] carrying a defined cargo of lipids, RNAs and proteins [214]. Reports also indicate a direct miRNA transfer between co-cultured macrophages and hepato-carcinoma cells via gap junctions [215]. Since gap junctions also exist between microglia and neurons [216], it would be interesting to investigate whether miRNAs can be transferred between these cells in a similar way. In addition, extracellular miRNAs can be endocytosed by target cells and bind to intracellular TLR7 to activate downstream signalling pathways [217]. Moreover, it was recently shown that microglia-derived let-7 leads to ethanol-induced neurotoxicity by activating neuronal TLR7 in rat brain slices [218]. These findings prompted the idea of “miRceptors” [219], and it would be interesting to investigate whether other known receptors can function as miRceptors. In summary, miRNAs released by glia may function as endocrine, paracrine and/or autocrine regulators and they may be endocytosed by target cells to modulate cellular function. Such an endocrine function has already been described for miRNAs from hypothalamic neural stem cells (hNSCs). Exosomal miRNAs from hNSCs were found circulating in the CSF of young mice, but several of these miRNA species were significantly reduced in the CSF of aged mice [220]. Subsequent treatment of aged mice with exosomal miRNAs from young mice resulted in an anti-ageing effect and an associated decrease in inflammatory mRNAs, such as TNF, IL-1β and IL-6.

Uptake of released miRNAs by nearby cells was previously observed when oligodendroglial exosomes carrying miRNAs could be endocytosed by neurons to improve their viability under stress conditions [221]. Additionally, exosome-mediated miRNA transfer to neurons has been shown when haematopoietic cells enter the brain under inflammatory conditions and release exosomes that are taken up by Purkinje cells [222]. In the context of ALS, transfer of miRNAs to motor neurons has so far been shown only from astrocytes [192]. In this study, exosomal miRNAs released by induced astrocytes from ALS patients with *C9orf72* mutations affected the maintenance and survival of wild-type mouse motor neurons. In a different study, it was shown that glia-to-neuron shuttling of bioactive miRNAs is possible via extracellular vesicles and that this transfer is responsible for gene expression changes within receiving neurons [223]. Pro-inflammatory activation of microglia resulted in the upregulation and release of miR-146a-5p in extracellular vesicles, which then fused with the neuronal membrane of cultured hippocampal neurons to transfer their miRNA cargo inside the cells. This resulted in a downregulation of *Syt1* and *Nlg1*, which are targets of miR-146a-5p, in the cell body and proximal dendrites, accompanied by a reduction in dendritic spine density and miniature synaptic currents.

Evidence for microglial miRNAs targeting neurons has also been proposed for other neurodegenerative diseases. For example, increased levels of let-7b have been found in the CSF of AD patients and it was shown to cause neurodegeneration by targeting neuronal TLR7 in AD mouse models [224]. Given that let-7 upregulation is also observed in activated microglia of SOD1 transgenic mice [107], it is not unlikely that its neurodegenerative effects are exerted in a similar way in ALS. Furthermore, depending on the identity of the miRNA released by microglia, its transfer into neurons can also be neuroprotective. For example, following a traumatic brain injury (TBI), there is an acute inflammatory response that persists chronically, including a pro-inflammatory activation of microglia, ultimately leading to neurodegeneration [225]. These microglia upregulate certain miRNAs, including miR-124-3p, both inside the cells and in released exosomes [226]. Treatment of injured cultured neurons with microglia-derived exosomes containing miR-124-3p results in inhibition of the inflammatory response by promoting IL-10 expression and suppressing IL-1β, IL-6 and TNF expression as a result of reduced mTOR (mammalian target of rapamycin) signalling. This also restores the number of neurite branches and total neurite length by suppressing the expression of neurodegenerative proteins such as phosphorylated Tau and amyloid-β peptide [226]. These effects are paralleled in vivo, with additional improvement in neurologic outcome in mice with TBI. On the other hand, downregulation of miR-124-3p in microglial exosomes has the opposite effect [226].

**Fig. 1:**
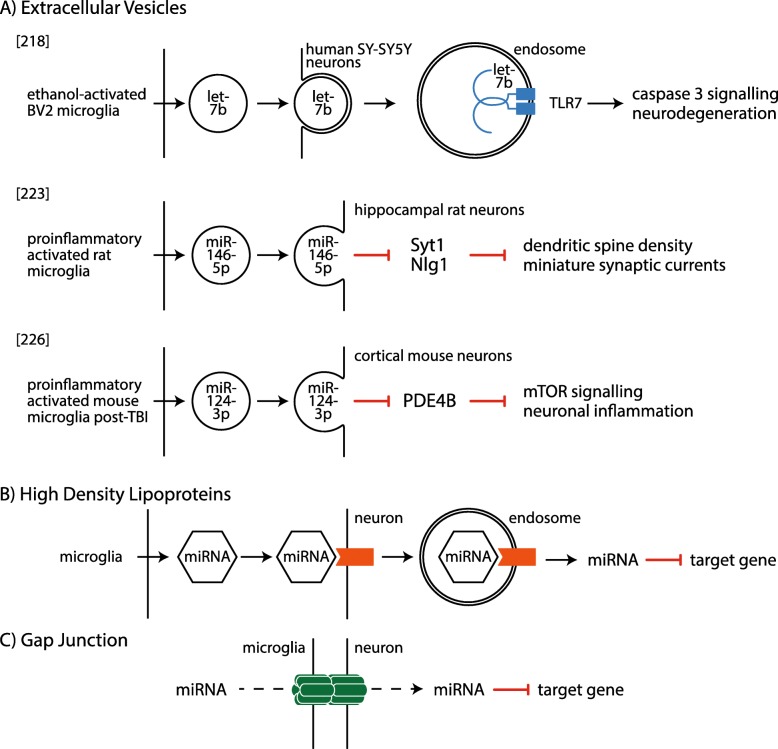
Different routes via which microglia-derived miRNAs could affect gene expression in neurons. Of these different mechanisms, transfer via extracellular vesicles (represented by a circle; **a**) is the best characterised transfer route between microglia and neurons. This has been shown through let-7b binding to TLR7 receptors (blue) within endosomes (double line circle) to cause neurodegeneration, miR-146-5p binding to target genes in hippocampal rat neurons to alter synaptic excitability properties, and miR-124-3p inhibiting mTOR signalling and activating a neuronal inflammation phenotype. Alternatively, miRNAs could affect gene expression in neurons through interactions with high-density lipoproteins (HDL; hexagon; **b**). These miRNAs released by microglia bound to proteins in HDL particles are endocytosed into the cell when binding to HDL receptors (orange) on neurons. These miRNAs are then released and regulate target genes. Alternatively, miRNAs could be directly transferred between microglia and motor neurons through gap junctions (green; **c**) where the two cell types are directly connected through a channel

The aforementioned studies provide evidence that microglia release miRNAs that can influence neighbouring neurons in the context of neurodegeneration, and although similar findings have been observed with other glial cell types in ALS, there is a gap in our knowledge about the role of microglia-derived miRNAs in ALS pathology. It is plausible that miRNAs of microglial origin are transferred to motor neurons and elicit functional changes relevant to neurodegeneration and/or neuroprotection in ALS (Fig. 1). This is particularly relevant to miRNAs that have been dysregulated due to a pro-inflammatory activation of microglia, or even those released by the transcriptionally distinct DAM population. To examine this, it would be necessary to determine which miRNAs are released by microglia and then validate their target genes in motor neurons. Moreover, it would be interesting to investigate whether these miRNAs correspond to those detected in the CSF and blood of ALS patients. This could be done by examining whether microglial markers are present on exosomes containing the circulating miRNAs. Such experiments would be the first step towards understanding whether microglia-mediated neurodegeneration in ALS involves the release of miRNAs.

## Microglia and associated miRNAs as therapeutic targets

Given the evidence of microglial activation as well as an altered miRNA expression profile in ALS patients and mouse models, they both appear as likely candidates for the development of therapeutic strategies. Many studies have explored the inhibition of specific microglial pro-inflammatory cytokines as a therapeutic strategy; however, this has so far proven unsuccessful (for a review see [227]). This is likely because of an absence of temporal and spatial specificity, but also due to simultaneously targeting the beneficial anti-inflammatory microglial factors. Indeed, it was previously shown that during disease progression, in addition to neurotoxic factors, ALS microglia concurrently show induction of neuroprotective factors such as insulin-like growth factor 1 (IGF1), progranulin (GRN) and triggering receptor expressed on myeloid cells 2 (TREM2) [87]. These responses are likely mediated by extrinsic regulatory factors such as signals released by dying motor neurons. In line with this, reducing proliferating microglia in ALS mice has no effect on survival [228], hence reflecting the multifaceted role of microglia in the disease. Therefore, neutralising the effect of activated microglia may not be a suitable therapeutic avenue.

Instead, miRNA modulation may be a promising strategy when used with the appropriate gene therapy tools, since a single miRNA can target several proteins involved in a signalling pathway associated with the disease. For example, miRNA knockdown may be used to reduce the expression of an upregulated miRNA. This can be done either by genomic editing, by targeting the transcription or processing of the miRNA, or by the use of antagomirs, which are chemically engineered oligonucleotides complementary to miRNAs; the use of locked nucleic acids in this last approach has technical benefits. By hybridising to miRNA and thus preventing its binding to the target mRNA, antagomirs allow normal translation of the target mRNA. On the other hand, downregulated miRNAs can be replaced with miRNA mimics that have the same sequence as the miRNA and thus bind to the mRNA targets to repress their translation. However, a problem with using miRNA mimics is the possibility of off-target effects because of the large number of mRNAs modulated by a single miRNA. Nevertheless, miRNA-based therapies have been tested in human clinical trials for other diseases, such as hepatitis C viral infection [229] and cancer [230], although, for neurodegenerative diseases, the challenge is the delivery of these reagents into the CNS and to the target cells. Finally, reprogramming ALS microglia towards a beneficial phenotype associated with the release of neuroprotective miRNAs may be another possible therapeutic avenue. In fact, redirecting microglia from a neurotoxic to a pro-regenerative phenotype has already been achieved by drugs targeting cell metabolism (reviewed in [231]), and is one of the current pharmacological approaches for the treatment of neuroinflammatory diseases associated with microglial activation, such as multiple sclerosis.

## Conclusion

In conclusion, given the evidence of an altered miRNA expression profile in ALS patients, as well as the presence of inflammation and the associated microglial reactivity, microglia are a likely source of dysregulated miRNAs, which are potential mediators of neurodegenerative processes. Furthermore, as miRNA dysregulation may be involved in the mechanisms of neurodegeneration and since a single miRNA can affect the expression of several genes, their modulation could change cellular phenotypes, thus representing a potential target for therapeutic intervention in the hopes of attenuating some of their detrimental functions and improving disease outcomes. Finally, although minimally invasive diagnostic tools and effective therapeutics for most CNS diseases are lacking, miRNAs associated with immune cells are promising candidates both for the development of biomarkers and treatments. Full understanding of the microglia-associated miRNA regulation/dysregulation and release is therefore crucial towards a comprehensive understanding of their role in ALS pathology and their therapeutic potential.

## Supplementary information

**Additional file 1.** List of miRNAs dysregulated in ALS. Dysregulated miRNAs in plasma, serum, cerebrospinal fluid (CSF), spinal cord tissue, skeletal muscle tissue, microglia and other cell types in ALS patients and animal models.

**Additional file 2. **List of genes and biological processes targeted by dysregulated miRNAs. List of genes targeted by the six miRNAs found concurrently upregulated in the biological fluids of ALS patients and inside microglia from *SOD1*^G93A^ mice. These are only the genes that have been experimentally validated to be targeted by each miRNA. Gene ontology analysis of these genes revealed enrichment in several biological processes, including processes commonly affected in ALS.

## Data Availability

Not applicable.

## Abbreviations

AD: Alzheimer’s disease
ALS: Amyotrophic lateral sclerosis
AP-1: Activator Protein 1
BzATP: 2′-3′-O-(benzoyl-benzoyl) ATP
C9orf72: Chromosome 9 open reading frame 72
CNS: Central nervous system
COX2: Cyclooxygenase 2
CREB: cAMP Response Element-Binding protein
CSF: Cerebrospinal fluid
DAM: Disease-associated microglia
eIF4F: Eukaryotic translation Initiation Factor 4F
ER: Endoplasmic reticulum
fALS: Familial amyotrophic lateral sclerosis
FTD: Frontotemporal dementia
FUS: Fused in sarcoma
GRN: Progranulin
HDL: High-Density Lipoprotein
IFN: Interferon
IGF1: Insulin-like Growth Factor 1
IL: Interleukin
LCA: Leukocyte Common Antigen
LFA: Lymphocyte Function-Associated molecule
MAPK: Mitogen-Activated Protein Kinase
MCP: Monocyte Chemoattractant Protein
MHC: Major Histocompatibility Complex
miRNA: MicroRNA
ncRNA: Non-coding RNA
NF-κB: Nuclear Factor kappa-light-chain-enhancer of activated B cells
NO: Nitric oxide
RNS: Reactive nitrogen species
ROS: Reactive oxygen species
sALS: Sporadic Amyotrophic Lateral Sclerosis
SOD1: Superoxide Dismutase 1
TARDBP: Transactive Response DNA-Binding Protein
TBI: Traumatic brain injury
TDP-43: Transactive Response DNA-binding Protein 43
TLR: Toll-Like Receptor
TNF: Tumour Necrosis Factor
TREM2: Triggering Receptor Expressed on Myeloid cells 2
